# Quasispecies Fitness Partition to Characterize the Molecular Status of a Viral Population. Negative Effect of Early Ribavirin Discontinuation in a Chronically Infected HEV Patient

**DOI:** 10.3390/ijms232314654

**Published:** 2022-11-24

**Authors:** Josep Gregori, Sergi Colomer-Castell, Carolina Campos, Marta Ibañez-Lligoña, Damir Garcia-Cehic, Ariadna Rando-Segura, Caroline Melanie Adombie, Rosa Pintó, Susanna Guix, Albert Bosch, Esteban Domingo, Isabel Gallego, Celia Perales, Maria Francesca Cortese, David Tabernero, Maria Buti, Mar Riveiro-Barciela, Juan Ignacio Esteban, Francisco Rodriguez-Frias, Josep Quer

**Affiliations:** 1 Liver Diseases-Viral Hepatitis, Liver Unit, Vall d’Hebron Institut de Recerca (VHIR), Vall d’Hebron Barcelona Hospital Campus, Passeig Vall d’Hebron 119-129, 08035 Barcelona, Spain; 2 Centro de Investigación Biomédica en Red de Enfermedades Hepáticas y Digestivas (CIBERehd), Instituto de Salud Carlos III, Av. Monforte de Lemos, 3-5, 28029 Madrid, Spain; 3 Biochemistry and Molecular Biology Department, Universitat Autònoma de Barcelona (UAB), Campus de la UAB, Plaça Cívica, 08193 Cerdanyola del Vallès, Spain; 4 Microbiology Department, Vall d’Hebron Institut de Recerca (VHIR), Vall d’Hebron Hospital Universitari, Vall d’Hebron Barcelona Hospital Campus, Passeig Vall d’Hebron 119-129, 08035 Barcelona, Spain; 5 Institute of Agropastoral Management, University Peleforo Gon Coulibaly, Korhogo BP 1328, Côte d’Ivoire; 6 Enteric Virus Laboratory, Section of Microbiology, Virology and Biotechnology, Department of Genetics, Microbiology and Statistics, School of Biology, University of Barcelona, 08028 Barcelona, Spain; 7 Enteric Virus Laboratory, Institute of Nutrition and Food Safety (INSA), University of Barcelona, 08028 Barcelona, Spain; 8 Centro de Biología Molecular “Severo Ochoa” (CBMSO, CSIC-UAM), Consejo Superior de Investigaciones Científicas (CSIC), Campus de Cantoblanco, 28049 Madrid, Spain; 9 Department of Clinical Microbiology, Instituto de Investigación Sanitaria-Fundación Jiménez Díaz University Hospital, Universidad Autónoma de Madrid (IIS-FJD, UAM) Av. Reyes Católicos 2, 28040 Madrid, Spain; 10 Biochemistry Department, Vall d’Hebron Institut de Recerca (VHIR), Vall d’Hebron Hospital Universitari, Vall d’Hebron Barcelona Hospital Campus, Passeig Vall d’Hebron 119-129, 08035 Barcelona, Spain; 11 Medicine Department, Universitat Autònoma de Barcelona (UAB), Campus de la UAB, Plaça Cívica, 08193 Bellaterra, Spain

**Keywords:** quasispecies, deep-sequencing, variability, rare haplotypes, fitness, mutagens

## Abstract

The changes occurring in viral quasispecies populations during infection have been monitored using diversity indices, nucleotide diversity, and several other indices to summarize the quasispecies structure in a single value. In this study, we present a method to partition quasispecies haplotypes into four fractions according to their fitness: the master haplotype, rare haplotypes at two levels (those present at <0.1%, and those at 0.1–1%), and a fourth fraction that we term *emerging haplotypes*, present at frequencies >1%, but less than that of the master haplotype. We propose that by determining the changes occurring in the volume of the four quasispecies fitness fractions together with those of the Hill number profile we will be able to visualize and analyze the molecular changes in the composition of a quasispecies with time. To develop this concept, we used three data sets: a technical clone of the complete SARS-CoV-2 spike gene, a subset of data previously used in a study of rare haplotypes, and data from a clinical follow-up study of a patient chronically infected with HEV and treated with ribavirin. The viral response to ribavirin mutagenic treatment was selection of a rich set of synonymous haplotypes. The mutation spectrum was very complex at the nucleotide level, but at the protein (phenotypic/functional) level the pattern differed, showing a highly prevalent master phenotype. We discuss the putative implications of this observation in relation to mutagenic antiviral treatment.

## 1. Introduction

Viral quasispecies [1] are intrinsically dynamic entities, with new genomes (haplotypes) being created during each replication cycle, mainly produced by the activity of an error-prone viral polymerase. The fate of each genome depends on its fitness [2]; that is, its capacity for replication in competition with other genomes in the quasispecies within the current environment. At a given time, the approximate fitness of a viral genome in a specific sample can be inferred from its frequency in the quasispecies [3,4]. That is, by the relative number of molecules belonging to the genome at that time point expressed as a fraction of the total. These frequencies can be considered a summary of the current molecular state of the quasispecies. The information gained by monitoring the molecular status of a viral quasispecies in an infection is particularly useful for following the viral response to a monoclonal antibody [5,6] or mutagenic agent [7,8]. Changes in the haplotype frequencies indicate the type of effects produced.

To characterize a viral quasispecies, we had to find an optimal balance between an analysis with high final coverage of shorter genomic fragments or with lower final coverage of larger fragments. The emergence of single-molecule real-time (SMRT) sequencing has opened the possibility to sequence large fragments, including entire viral genomes, in a single read as has been reported in influenza virus [9], hepatitis C virus [10], and human immunodeficiency virus [11]. However, SMRT technology typically has lower coverage (maximum 4M reads per run) and higher error rates than Illumina techniques [12,13]. SMRT is proven to have great value for assembling genomes [14] but requires further development to be useful for quasispecies analysis. For the present study, we needed very extensive final coverage (≥10^5^ reads) to limit potential bias caused by a small sample size [3]. Hence, we used the MiSeq Illumina instrument, which enables analysis of amplicons in a size range of 300–500 base pairs (bp) at very high final depth and with acceptable error levels. The methods we propose are independent of the sequencing platform used. The only requirement is to have a set of high-quality haplotypes with their frequencies in the quasispecies. For this purpose, the sequencing data obtained here were treated to preserve the integrity of an amplicon’s full-length reads; reads were either accepted or refused attending to their quality; however, they were never trimmed, except for the primers. Thus, the term *haplotype* used here refers to amplicon haplotypes, not to viral haplotypes, and the analyses are done amplicon-to-amplicon or on a single amplicon.

In a previous publication [7], we introduced the term *rare haplotype load* (RHL) in the context of a controlled experiment with HCV-infected hepatocytes treated with two mutagenic agents, the nucleoside analogues ribavirin (1-*β*-D-ribofuranosyl-1-*H*-1,2,4-triazole-3-carboxamide) and favipiravir (T-705; 6-fluoro-3-hydroxy-2-pirazinecarboxamide), and one inhibitor, sofosbuvir (isopropyl (2S)-2-[[[(2R,3R,4R,5R)-5-(2,4-dioxopyrimidin-1-yl)-4-fluoro-3-hydroxy-4-methyl-tetrahydrofuran-2-yl]methoxy-phenoxy-phosphoryl]amino]propanoate) [7]. The RHL refers to the fraction of genomic molecules in the quasispecies representing low- to very low-fitness haplotypes, and it was used as a biomarker of mutagenesis. In this study, we extend the concept of quasispecies partitioning according to their fitness (frequency within the population) into four fractions or haplotype categories: the master or dominant haplotype (present at the highest frequency within the quasispecies); the RHL divided into two levels, haplotypes present at less than 0.1% and haplotypes present from 0.1% to 1%; and a fourth category including emerging haplotypes, defined as those present at frequencies below the master value and above 1%. Emerging haplotypes are considered to have the potential to proliferate, attain higher frequencies, and possibly overtake the current master. 

Here, we propose to represent quasispecies evolution as the changes observed in the volume (fraction of molecules) of the four quasispecies fitness fractions (QFF) combined with the Hill number profile (HNP) [15], which quantifies the effective number of haplotypes. This combination provides a means to visualize and analyze molecular changes in the composition of a quasispecies over time. Three data sets were used for this purpose: (1) a technical clone of the complete SARS-CoV-2 spike gene [16], sequenced using 12 overlapping amplicons; (2) a subset of data previously used in a study of the RHL as a marker of lethal mutagenesis [7]; and (3) data from a clinical follow-up study of a patient chronically infected with hepatitis E virus (HEV) and treated with ribavirin (RBV), reported here for the first time. 

## 2. Results

**Figure 1:**
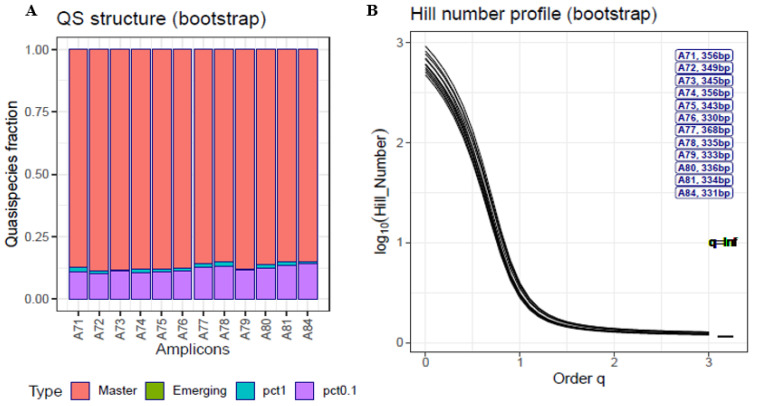
Quasispecies fitness fractions (**A**) and Hill number profile (HNP) plots (**B**) for a SARS-CoV-2 technical clone. The labels on the right of the HNP are sorted top-down in decreasing order of Hill number at q = 2; the ARTIC amplicon nomenclature is used (A71 to A84). Pct1 low fitness (0.1 < Freq ≤ 1%), pct0.1 very low fitness (Freq ≤ 0.1%). Each bar or curve corresponds to an amplicon of the S gene. Bootstrap values are represented. Coverage range: 51,102–299,349 reads; median 76,954 reads.

The technical clone is the simplest example (Figure 1). Despite its name, the commercial product carries multiple errors (although at low levels) due to the chemical yield at each synthesis step, which by definition never reaches 100% [17]. The errors observed include mainly deletions, which in the present case were bioinformatically corrected, but there were also some point mutations, possibly carry-overs from previous synthesis steps, which were left as they were. Some of these errors might also have been caused during the PCR amplification or sequencing steps. In Figure 1A, the QFF plot shows the <0.1% fraction which has a median of 0.11 (interquartile range [IQR] 0.02), the 0.1% to 1% fraction with a median of 0.011 (IQR 0.0058), no emerging haplotypes, and the master haplotype with a median of 0.876 (IQR 0.023). The HNP (Figure 1B) curve shows a steep decrease from q = 0 to q = 1. The curves remain asymptotically flat from q = 1.5 onwards, and there is only a small difference from q = 3 to q = ∞. 

**Figure 2:**
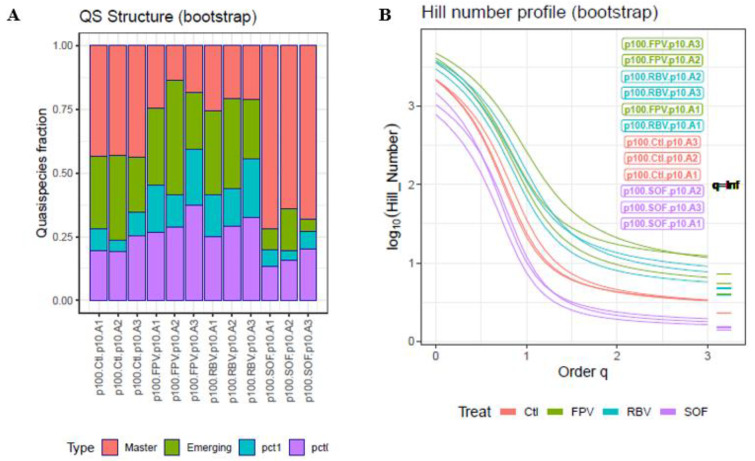
QFF (**A**) and HNP (**B**) plots for the HCV dataset. The labels on the right of the HNP are sorted top-down in decreasing order of Hill number at q = 2. Ctl control; FPV favipiravir; RBV ribavirin; SOF sofosbuvir. A1 to A3 refer to the amplicons analyzed. Pct1 low fitness (0.1 < Freq ≤ 1%), pct0.1 very low fitness (≤0.1%). Bootstrap values are represented. Coverage range 48,057–335,535 reads; median 206,354 reads.

The controlled experiment with cultured HCV-infected human hepatoma cells treated with mutagens or inhibitors yielded richer plots (Figure 2A), which are characteristic of the effects of treatment. The median QFF and IQR values of the three amplicons for each fraction and under each condition are given in Table 1. Inhibitor treatment resulted in lower fractions of emerging haplotypes and RHL haplotypes with respect to the control, as well as higher master frequencies. In contrast, mutagen exposure yielded higher RHL fractions and a depressed master relative to the control. In Figure 2B, the HNP is shown with labels sorted top-down in decreasing order of Hill number at q = 2. As the lower QFFs increase in volume and the master volume decreases, the curves remain at higher levels, and show a larger difference between q = 3 and q = ∞. Some curves cross over each other. This generally happens when there is one quasispecies with a large number of haplotypes having limited diversity in the frequencies, and a second with a smaller number of haplotypes but with higher diversity in the frequencies. 

**Figure 3:**
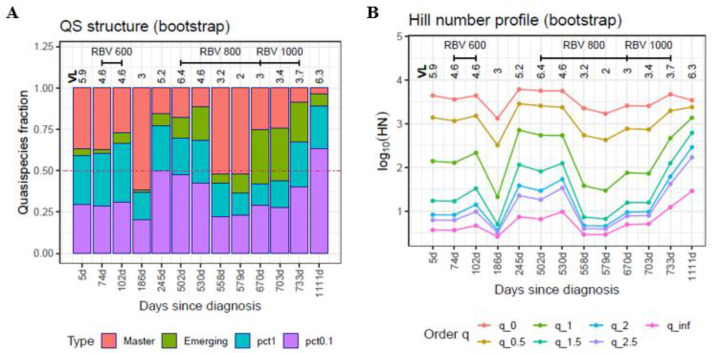
QFF (**A**) and HNP (**B**) plots for the HEV patient follow-up. The HNP is plotted here as cross-sections of the profile at given q values. Each line corresponds to a q value; the lines show how this value changes over time. Bootstrap values are represented. On the x-axis, days since the diagnosis for each sample. Coverage range: 53,307–503,770 reads; median 328,271 reads.

The changes occurring in the viral quasispecies following sequential RBV administration in the clinical case of HEV infection are shown in Figure 3A. In the first sample (baseline), analyzed on day 5 after the diagnosis (23 May 2018), the viral quasispecies already had a high burden of rare haplotypes at both <0.1% and 0.1–1%, with the master haplotype at <50%. The first mutagenic treatment (RBV 600mg) increased the RHL while reducing the master haplotype. Treatment was stopped on day 158 following the diagnosis, and 28 days later (20 November 2018), the master haplotype predominated in the quasispecies, but at a low viral load (3 logs).

Two months later (245 days since diagnosis, 18 January 2019), in the absence of treatment, the quasispecies had diversified to a higher level than was seen at baseline, with the 0.1% RHL reaching 50%. Nine months later (502 days since diagnosis, 2 October 2019), the viral load had increased to more than six logarithms, and a larger RBV dose (800mg) was prescribed. One month later (530 days since diagnosis, 30 October 2019), the quasispecies showed a structure very similar to that of the previous analysis in QFF terms, but the viral load had decreased by 2 logs. Suddenly, one month later (558 days since diagnosis, 27 November 2019) while the patient was still under treatment, the master sequence recovered to >50%, and remained at the same level for an additional 20 days (day 579 since diagnosis, 18 December 2019), whereas the viral load decreased to 2 logs. Three months later (670 days since diagnosis, 18 March 2020), the volume of emerging haplotypes had increased while the master haplotype showed a >50% decline with respect to the previous time point, with slightly higher viral loads. This structure was maintained for another month, at similar viral loads. Finally, one month later (733 days since diagnosis, 20 May 2020), when the master haplotype had further declined to <10%, treatment had to be stopped. One year later (1111 days since diagnosis, 2 June 2021), in the absence of treatment, the master haplotype was present at 3.6%, the emerging volume was 7.9%, and >88.5% of the quasispecies was comprised of haplotypes with frequencies < 1%. The HNP (Figure 3B) shows how the Hill numbers, at selected q values, changed over time.

**Figure 4:**
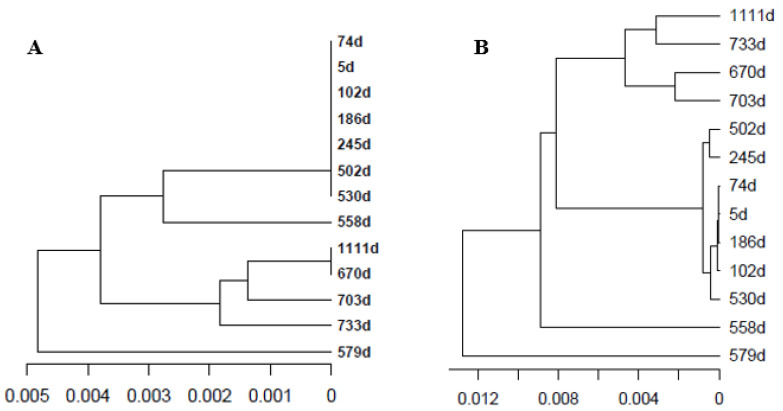
UPGMA tree of the master haplotypes based on raw nucleotide distances (**A**), and quasispecies tree based on the Da population distances (**B**) taking the top 50 haplotypes in each sample. Each sample is labeled as days since the diagnosis.

The UPGMA tree of the master sequences of all samples (Figure 4A) shows that the same master haplotype was maintained from the time of the diagnosis up to 530 days later (30 October 2019). From then on, the master differed at each time point except the last one (1111 days since diagnosis, 2 June 2021) when the master was the same as the sample at 670 days (18 March 2020). The UPGMA tree (Figure 4B) resulting from the Da population distances based on the top 50 haplotypes in each sample, displays a similar structure. The samples at 558 and 579 days since diagnosis (27 November 2019 and 18 December 2019) show a divergence in the structure and in the master sequence. These correspond to the lowest viral loads in the follow-up, with the master sequence predominating in the quasispecies.

**Figure 5:**
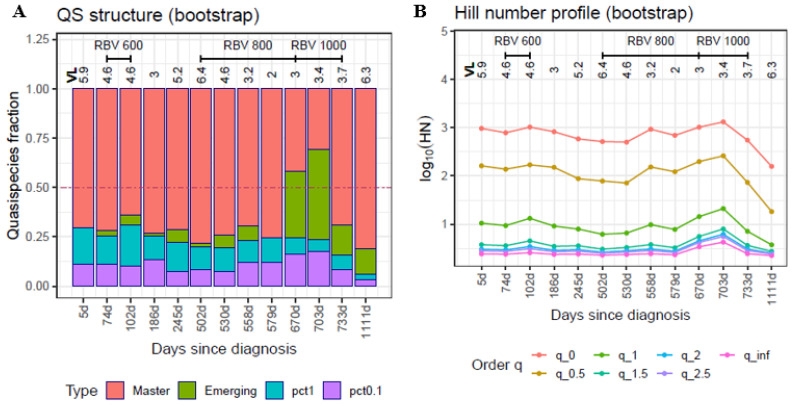
QFF (**A**) and HNP (**B**) plots for the quasispecies as amino acid haplotypes (phenotypes) for the HEV follow-up. Viral loads (VL) are expressed as logarithms. RBV, ribavirin. Pct1 low fitness (0.1 < Freq ≤ 1%), pct0.1 very low fitness (≤0.1%).

To understand why the profile of the last sample (1111 days since diagnosis, 2 June 2021), analyzed after one year with no treatment, showed a highly mutagenized quasispecies, the nucleotide haplotypes were translated to amino acids and recollapsed to obtain amino acid haplotypes (phenotypes) with their corresponding frequencies (Figure 5A,B). The last sample (1111 days since diagnosis, 2 June 2021) shows a master phenotype accounting for >80.9% of the molecules. The master phenotype clearly predominated in the quasispecies along treatment, except in two samples (at end of treatment with RBV 800 mg and during treatment with RBV 1000 mg), where there was a change in the predominant amino acid sequence (Figure S1a). At the other time points, the same master sequence predominated in the quasispecies. These findings indicate that although a quasispecies can have a highly mutated spectrum at the nucleotide level, it may remain almost unchanged at the amino acid level, suggesting that functionality is at least transiently maintained (Figures S1a,b and S2).

**Figure 6:**
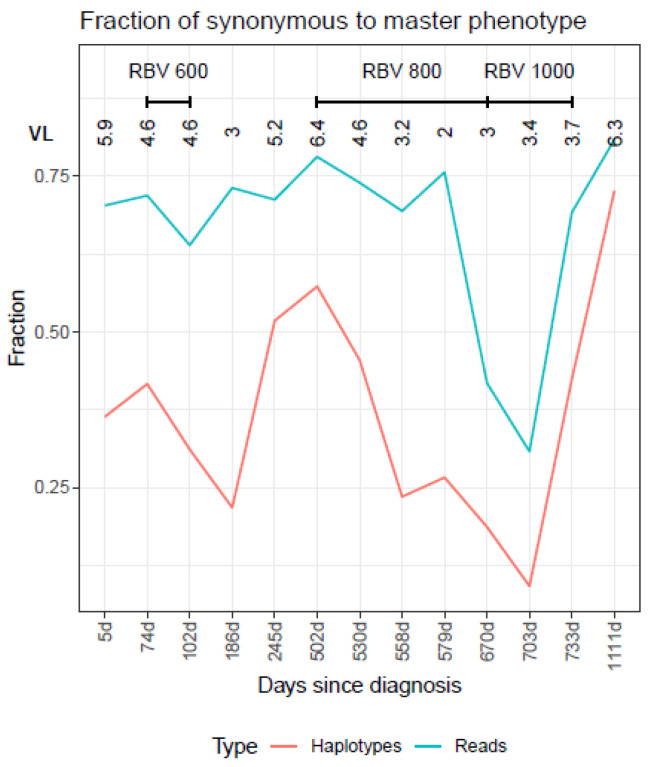
Fraction of haplotypes synonymous to the master phenotype (orange), and fraction of reads for these haplotypes (green). On the x-axis, days since the diagnosis for each sample. Viral loads (VL) are expressed as logarithms. RBV, ribavirin.

The number of synonymous haplotypes corresponding to the master phenotype steeply increased after the treatment discontinuations (Figure 6). That is, the various haplotypes identical to the master phenotype generated during treatment were able to easily increase in frequency when it was stopped, as they all had highest functional fitness. 

A UPGMA tree of master phenotypes based on their Grantham amino acid distances, and a quasispecies tree based on Da population distances were constructed using the top 20 most frequent phenotypes in each sample (Supplementary Figures S1a,b and S2). Apparently, viral treatment led to production of a rich set of haplotypes synonymous to the master phenotype, all with equal or comparable fitness, which proliferated when treatment was discontinued. This was evidenced by the decline in the master haplotype volume and the increase in emerging haplotypes synonymous to the master. As a whole, the resulting quasispecies might be more resistant to further mutagenic treatment and better fit to its current environment. A large number of highly fit haplotypes could correspond to a large number of molecular pathways to escape a treatment.

A final illustration of this conclusion is provided in Supplementary Figures S3 and S4, where Montserrat plots depict the distribution of the 1000 most abundant haplotypes and all phenotypes in the last sample (1111 days since diagnosis, 2 June 2020). In Montserrat plots, haplotypes are sorted by number of substitutions with respect to the master first, and by decreasing order of abundance second [18]. Each successive peak represents additional differences with respect to the master. In correspondence, Supplementary Figure S5 shows the mean number of differences with respect to the master haplotype/phenotype per read, at both the nucleotide level (substitution load) and amino acid level (mutation load). Notably, the ratio of the nucleotide substitution load to the amino acid mutation load was 13.31 in the last sample (one year without treatment), only 1.24 in the baseline sample, shortly after diagnosis, and 6.57 when the last treatment was discontinued. In addition, the mutation load (amino acid level) in the last sample was the lowest value in the series, despite the highly diverse quasispecies.

## 3. Discussion

The presence of a broad repertoire of genomes in the mutant spectra of RNA viruses represents a challenge for annotating and describing the genomes and the neighbor relationships among them. Several procedures have been developed to rank subgroups of related sequences within mutant spectra [19,20,21,22,23]. Alternative approaches have monitored mutant spectrum composition and diversification through quantification of diversity indices [4,24] and haplotype mapping using two-dimensional neural networks [25,26].

In our previous studies we introduced a number of new diversity indices, which, when adequately combined, provided information on both the abundance and divergence of haplotypes within a viral population [4]. In the present report, we have gone one step further, creating a procedure to divide a quasispecies population into four fractions using a fitness partition (QFF). The advantages of this approach include better statistical properties than most diversity indices and inclusion of the biological features of the population. The QFF procedure goes beyond a previous proposal restricted to the two fractions at lowest frequency in the mutant ensemble [7]. In addition, we used the Hill number profile [15] to provide a complementary view of the quasispecies structure. The profile is an enriched summary that assigns increasing weights to the haplotype frequencies in the quasispecies as the order q increases. In addition, the units provided (number of equally fit haplotypes) contribute to the interpretation of the results.

Bootstrapping provided an efficient down-sampling of mutant distributions in the quasispecies samples to the minimum coverage, thereby allowing comparisons regardless of the sample size. Hill numbers of orders below two are highly dependent on sample size and must be corrected. However, it is advisable to correct all diversity values. QFF is compatible with other ranking procedures used to study quasispecies, as the proposed fractioning can be applied to genome subsets obtained by other means. In particular, it can be a useful complement to self-organized fitness maps based on artificial neural networks [26]. It may also provide support to precisely define the SARS-CoV-2 mutant spectra which, according to recent results, are populated by a large proportion of low-frequency haplotypes [27,28].

The rationale behind the QFF definition and its main contribution to quasispecies analysis resides in the biological meaning of each fraction. Observation of a significant fraction of molecules corresponding to very low fitness haplotypes is only consistent with the presence of a mechanism that can generate mutants at a high rate, but cannot increase their frequency relative to competing genomes. Furthermore, concerning the response of HEV to RBV, the course of any treatment giving rise to resistant variants is only consistent with a decline in volume of the master haplotype together with a parallel increase in emerging haplotypes.

We used both the QFF and HNP to visualize and analyze two simple case models: the first, a SARS-CoV-2 technical clone, and the second, populations from a controlled experiment in which a clonal HCV population was serially passaged in cultured human hepatoma Huh-7.5 cells. These two datasets were used to demonstrate the capability of the proposed QFF/HNP combined method. Finally, the procedure was applied to a complex clinical case: follow-up of an HEV-infected patient treated with various RBV doses with two discontinuations, to illustrate its performance for clinical purposes. As this is a single example, the discussion provided for this case should be considered explanatory of how the information obtained with the QFF procedure can be of clinical value.

Lethal mutagenesis is a useful antiviral approach that consists of driving viral genomes to extinction—pushing the virus to cross the error catastrophe threshold by increasing the viral mutation rate above the maximum level compatible with infectivity—without mutagenizing the host cells [29,30,31]. A recent example of successful application of lethal mutagenesis is the use of molnupiravir against COVID-19 [32]. Several other cases have been reviewed [8]. Currently, no specific drugs have been approved for HEV infection; RBV is the main option as an off-label drug. RBV is a broad-spectrum mutagenic agent [33,34,35] that can increase mutation rates and result in extinction of the virus by lethal mutagenesis [36]. However, during RBV treatment, a reduction in the effective antiviral dose by low adherence or early treatment interruption can allow residual viral replication and production of rescue variants with decreased RBV sensitivity or altered replication fitness [37,38,39]. This could lead to selection of mutations resistant to RBV and the appearance of resistant variants.

In our study, deep-sequencing of samples from an HEV-infected patient receiving RBV treatment showed that the mutagenic agent led to a reduction in the most highly represented sequence (master) at the nucleotide level, together with a significant increase in the number of rare haplotypes, findings in agreement with a mutagenic effect of RBV on this virus. When the first round of RBV treatment (600 mg) was stopped, HEV relapsed, showing an increase in viral load and re-acquisition of the master sequence that had predominated before treatment was started. In the second and third rounds of RBV treatment, with increases in the drug concentration to 800 mg and 1000 mg and evidence of good adherence to therapy, the viral load remained unchanged and even increased, while the master sequence decreased once again. Notably, after stopping 1000 mg RBV treatment, the frequency of the master genome declined even further despite a three-log viral load increase. 

However, when we examined the quasispecies at the protein (phenotypic/functional) level, the pattern drastically changed, showing a highly predominant master phenotype, despite the complexity of the mutation spectrum observed at the nucleotide level. On stopping treatment, the synonymous variants generated had the same replication capacity as the original master genome and were able to proliferate, leading to a high diversity of genomes that could all express the same phenotype. The reason why the dynamics of the mutant spectrum involved haplotypes with silent mutations is unknown. Furthermore, we cannot exclude that some of the variants produced might have had lower sensitivity to RBV. Both these reasons; a large reservoir of functional genomes and decreased sensitivity to treatment could explain the viral load increase in the presence of a high dose of mutagen. Failures in HEV RBV treatment have been described, but detection of RBV-resistant mutations requires sequencing the full HEV genome [38,39,40]. In our case, we assumed that the effects of RBV on the sequenced amplicon would be similar to the impact on the remainder of the genome, as the virus cannot drive the mutagenic effects. Future approaches using the new SMRT circular consensus sequencing technology may provide further support of this amplicon to genome relationship. 

The mutagenic effect of RBV on HEV could lead to viral extinction, but it also involves a risk of accumulating advantageous mutations and selecting fitness-enhancing ones [36]. In our study, while the patient was receiving antiviral treatment, a number of synonymous haplotypes were produced, which as a whole seemed to be stronger against further treatment and improved accommodation of the quasispecies to its current environment. The findings from our patient suggest that mutagenic antiviral therapy should ideally be combined with other antivirals. When this is not possible, as in HEV infection, treatment should be maintained, even after serum RNA tests negative, to avoid relapses which could lead to selection of fitness-enhancing mutations and treatment failure. 

The QFF approach, although simple and straightforward, has some technical limitations, mainly due to the current state of high-throughput sequencing technology. The sequence length and error level are two aspects that limit each other. We can sequence amplicons up to slightly more than 500 bp in length with an acceptable error level using paired-end technology, but we cannot sequence full-length viral genomes of a few thousand base pairs at high depth (10^4^–10^5^) with low error levels and high coverage. In this study, we used amplicons larger than 300 bp and assumed that either the amplicon underwent effects similar to those that would occur in the whole genome, or that the amplicon was the target to study. Another limitation relates to a factor observed in all -*omics*, where the experimental design is of the utmost importance to avoid bias. Even in balanced designs, batch effects should be taken into account. In our case, we were not interested in a detailed account of point mutations and indels; rather, we aimed to provide a picture of the macroscopic structure of a quasispecies. This information is of value, as high-resolution deep sequencing unveils myriads of low frequency mutations that should correspond to an extensive repertoire of minority genomes [28]. In providing this general view, we can accept a certain presence of artifactual haplotypes, provided that all samples in the experiment show the same noise level, hence the need for an accurate experimental design. Filtering above the error level to avoid all artifacts would involve a considerable loss of information and jeopardize the type of analysis we are proposing. The impact of filtering all haplotypes below 0.1% or 1% on the total reads number, that is the information loss, can be seen in Figure 1, Figure 2 and Figure 3. Nevertheless, low-level filters of very few reads per haplotype (e.g., 1–10 reads at 1 × 10^5^ coverage) will have an impact on the number of haplotypes—that is, on the Hill numbers of low order (<1.5)—but will have a minimum effect on the number of reads. It could be helpful to perform a sensitivity analysis of the various diversity indices used with respect to this threshold. These limitations and warnings are equally applicable to any quasispecies study, whatever the indices or variables used, and are not exclusive to the methods proposed here.

We propose a simple method for monitoring the changes occurring in a quasispecies at the molecular level, involving fitness fractions and the Hill number profile. This combined method, which provides an easily interpretable visualization of quasispecies evolution in viral terms, was applied to two simple cases as a demonstration, and to samples from an RBV-treated HEV patient to illustrate its clinical value. The method is based on bioinformatic treatment of sequencing data to obtain a set of high-quality amplicon haplotypes with their corresponding frequencies to represent the quasispecies structure. Use of next-generation sequencing technology in combination with a good experimental design provides exceptional opportunities to study complex quasispecies and follow their evolution at the molecular level, in both research laboratories and clinical settings.

## 4. Materials and Methods

### 4.1. Samples

Two datasets were used to develop quasispecies molecular characterization with QFFs and the HNP:

- A technical clone of the SARS-CoV-2 *S* gene (Twist Synthetic SARS-CoV-2 RNA Control 2 MN908947.3, TWIST Biosciences, South San Francisco, CA, USA) sequenced in 12 amplicons [17]. Commercial Twist Synthetic SARS-CoV-2 RNA controls consist of six non-overlapping 5-Kb fragments generated from in vitro transcription of gene fragments. The synthetic controls were diluted at 1:10 to a concentration of 1 × 10^5^ copies per microliter, PCR-amplified following the Sub-ARTIC v3 protocol [41] using a set of 28 primers (A71 to A84) covering the full *S* gene, and sequenced on a MiSeq Illumina system [42]. The haplotypes and corresponding frequencies in this analysis included all haplotypes common to both DNA strands after a previous filter at 2 reads. That is, at a minimum of 2 + 2 reads.
- Three HCV amplicons from samples taken from a controlled experiment, in which HCV-infected human hepatoma cells were observed in the presence or absence of RBV, favipiravir, or sofosbuvir [43,44]. Briefly, HCV p0 was the parental viral population obtained by electroporation of a transcript of plasmid Jc1FLAG2(p7-nsGluc2A) (a chimera of J6 and JFH-1, genotype 2a) [45] into Huh-7.5-Lunet cells and amplification in Huh-7.5 cells [46]. HCV p100 resulted from passaging the HCV p0 population 100 times in Huh-7.5 reporter cells [47]. HCV p100 was subsequently passaged 10 additional times in the presence of favipiravir (T-705) (Atomax Chemicals Co., Ltd., Shenzhen, China), RBV (Sigma, Kawasaki, Japan), or sofosbuvir. Drug concentrations were adjusted to produce comparable inhibition of HCV p0 progeny production. The amplicons sequenced covered the following HCV genomic regions: A1, spanning genomic residues 7626 to 7962; A2, residues 7941 to 8257; and A3, residues 8229 to 8653. The haplotypes and corresponding frequencies in this analysis included all haplotypes common to both strands, with no previous abundance filter; that is, a minimum of 1 + 1 reads.

In addition, we describe the quasispecies findings from the clinical follow-up case of a 27-year-old patient who acquired chronic HEV infection after undergoing two kidney transplantations. The patient received three different RBV regimens (Table 2). First, 600 mg per day for 3 months, which led to a significant reduction in viral load without achieving undetectable HEV RNA. Treatment was stopped. The patient relapsed, and a second treatment with RBV 800 mg daily was prescribed, with a new reduction in HEV levels. At month 5, RBV dosage was increased to 1000 mg daily for two additional months. Treatment was discontinued because of a lack of antiviral response, and viral load jumped three logs at 10 days after stopping treatment. A single amplicon covering genomic positions 6323 to 6734 on the ORF2 region was sequenced. The haplotypes and corresponding frequencies in this analysis included all haplotypes common to both DNA strands after a previous filter at 2 reads. That is, a minimum of 2 + 2 reads.

### 4.2. Processing the Sequencing Data

The aim of the sequencing data treatment was to discard error-bearing reads while preserving full-length read integrity, so that haplotypes that completely cover the amplicon with their respective frequencies were incorporated. The steps in this process are the following:

- Obtain Fastq files with Illumina 2 × 300-bp paired-end reads;
- Recover full amplicon reads with FLASH [48] (min. 20-bp overlap, max. 10% mismatches). The 300-bp reads, when overlapped, result in reads covering complete ~400–500 bp amplicons;
- Remove full reads with 5% or more bases below a Phred score of Q30;
- Demultiplex and trim primers (max three differences accepted);
- Collapse reads (molecules) to haplotypes (amplicon-genomes) and their frequencies. The frequencies were calculated per haplotype of each amplicon;
- In certain cases, remove all haplotypes below a fixed frequency threshold;
- Remove all haplotypes that are not common to both DNA strands.

The final obtained haplotypes and their frequencies were the basis for all further calculations.

The SARS-CoV2 dataset consists of 12 amplicons (min 330 bp, max 368 bp, median 340 bp). The HCV dataset consists of three amplicons (312, 318, and 423 bp). Finally, the HEV study is based on single 363-bp amplicons (Supplementary Table S1). The amplicon sizes provided are the final result after primer trimming.

### 4.3. Quasispecies Fitness Partitions

At a given time, a quasispecies is usually comprised of a highly predominant haplotype, a few low- to medium-frequency genomes, various rare haplotypes with very low fitness but still able to replicate to some level, and some defective genomes unable to replicate. This composition can be modeled using the set of frequencies of all haplotypes as parameters of a multinomial distribution (Equation (1)),

(1) $$\Pi =\left\{p_{1},p_{2},\ldots ,p_{n}\right\}with\overset{n}{\underset{i}{\sum }}p_{i}=1$$

where *p*_1_, *p*_2_, …, *p_n_* represent the various haplotypes, arranged in order of decreasing frequency. The parameters, *p_i_*, are sorted in decreasing order without a loss of generality. In this way, the quasispecies can be partitioned into fractions limited by frequency thresholds of interest, as in Equation (2), where a partition into four fractions is illustrated,

(2) $$\begin{array}{c} \Pi_{1}=\left\{p_{1},p_{2},\ldots ,p_{k}\right\},\forall p_{i}:p_{i}\geq p_{k}\\ \Pi_{2}=\left\{p_{k+1},p_{k+2},\ldots ,p_{l}\right\},\forall p_{i}:p_{l}\leq p_{i}<p_{k}\\ \Pi_{3}=\left\{p_{l+1},p_{l+2},\ldots ,p_{m}\right\},\forall p_{i}:p_{m}\leq p_{i}<p_{l}\\ \Pi_{4}=\left\{p_{m+1},p_{m+2},\ldots ,p_{n}\right\},\forall p_{i}:p_{n}\leq p_{i}<p_{m}\\ {p^{\prime }}_{1}=\sum_{i}^{k}p_{i};{p^{\prime }}_{2}=\sum_{k+1}^{l}p_{i};{p^{\prime }}_{3}=\sum_{l+1}^{m}p_{i};{p^{\prime }}_{4}=\sum_{m+1}^{n}p_{i};with\sum_{i=1}^{4}{p^{\prime }}_{i}=1 \end{array}$$

and where, *p*′_1_, *p*′_2_, *p*′_3_, and *p*′_4_ represent the four fractions.

In the typical quasispecies structure mentioned above, the four fractions can be defined as follows:

- **Master**: the fraction of molecules belonging to the most frequent haplotype; that is, the one present at the highest percentage (*p*′_1_ = *p*_1_);
- **Emerging**: the fraction of molecules present at a frequency > 0.1% and less than the master percentage, belonging to haplotypes that are able to compete with the predominant one and possibly replace it (*p*′_2_);
- **Low fitness**: the fraction of molecules present at frequencies from 1% to 0.1%, belonging to haplotypes that have a low probability of progressing to higher frequencies (*p*′_3_);
- **Very low fitness**: the fraction of molecules present at frequencies < 0.1% belonging to haplotypes with very low fitness and to defective genomes. The likely fate of these molecules individually is degradation, but the fraction is continuously fed with new very low fitness genomes produced by replication errors or by host editing activities (*p*′_4_).

The evolutionary trends occurring in a viral quasispecies can be characterized by determining the changes that take place in the molecular volume of these fractions as a function of time.

The coefficient of variation (*CV*) of a proportion, *p*, for a given sample size, *N* (i.e., the standard deviation expressed in expected value units), is given in Equation (3). When the proportion is very small, it can be approximated by calculating the square root of the inverse of the product of the sample size multiplied by the proportion, as in Equation (4),

(3) $$CV\left[\frac{x}{N}\right]=CV\left(p,N\right)=\frac{sd\left(p,N\right)}{p}=\sqrt{\frac{\left(1-p\right)}{Np}}$$

(4) $$CV\left(p,N\right)\approx \sqrt{\frac{1}{Np}}$$

where *x* is the observed count, *N* is the sample size, and *p* is the observed proportion. In the experiments described in this study, the coverage (sample size, *N*) ranged from 10^4^ to 10^6^, with the average larger than 1 × 10^5^, and our aim was to observe haplotypes present in very low proportions (*p*); that is, <0.1% (<×10^−3^). Individually, haplotypes considered to have very low fitness will show a high *CV*, which means that some of them can be easily overlooked in a single sample. Nevertheless, when grouped together, as is seen in the above partition (the *p*′ in Equation (2)), they amount to a much higher proportion than when counted individually and become more statistically stable. Thus, the fraction of molecules in the quasispecies belonging to haplotypes having very low fitness, *p*′_4_, becomes more stable to sampling and less dependent on the sample size [7] than any single haplotype at these frequencies.

### 4.4. Hill Numbers

In addition to the partition described above, we can determine the diversity profile of a quasispecies with the use of Hill numbers [4,15,49]. Based on the expression of the generalized diversity index, of order *q*, *^q^H*(*p*) is given in Equation (5),

(5) $${}^{q}H\left(p\right)=\overset{H}{\underset{i=1}{\sum }}p_{i}^{q}$$

the Hill number of order *q*, *^q^D*, of a quasispecies corresponds to the number of equally fit haplotypes comprising a quasispecies with the same general diversity, *^q^H*, as the original quasispecies, as is shown in Equation (6).

(6) $$\overset{H}{\underset{i=1}{\sum }}p_{i}^{q}=\overset{D}{\sum }{\left(\frac{1}{D}\right)}^{q}=D{\left(\frac{1}{D}\right)}^{q}=D^{1-q}$$

This results in Equation (7),

(7) $${}^{q}D\left(p\right)={\left(\overset{H}{\underset{i=1}{\sum }}p_{i}^{q}\right)}^{1/\left(1-q\right)}$$

where *^q^D*(*p*) is the Hill number of order *q*, calculated from the haplotype frequencies observed in the quasispecies, *p_i_*. The diversity indices, *^q^D*(*p*), obey the replication principle [4] and are expressed in intuitive units (number of equally fit haplotypes). In ecology, the replication principle states that if we have *N* equally large, equally diverse groups (quasispecies), and no species (genomes) in common, the diversity of the pooled groups must be *N* times the diversity of a single group.

At increasing values of *q* starting from 0, we obtain diversity values that are also a transformation of other classical diversity indices:

- At *q* = 0, the Hill number is the number of haplotypes;
- at *q* = 1, it corresponds to the exponential of Shannon entropy;
- at *q* = 2, it is the inverse of the Simpson index; and
- at *q* = ∞, it is the inverse of the predominant haplotype.

The Hill number profile of a quasispecies is the curve we obtain from *q* = 0 to 3, plus the value at infinity. The curve becomes asymptotic beyond order 2 and reaches its minimum at infinity. As a result of the quasispecies values spanning a large range—more than three orders of magnitude—the Hill number profile is best represented in *log*_10_ units.

### 4.5. Abundance Filter Effect on Haplotype Distribution

The goal of step 6 in the sequencing data treatment described above is usually to limit technical errors (PCR + sequencing) to a level suitable for the purposes of the study, while maintaining the integrity of full amplicon reads. The required frequency threshold can be established through the use of clones that have been processed in parallel with the clinical samples and sequenced in the same run [50].

The immediate effect of this filter is removal of all haplotypes with abundances below the threshold, which in probabilistic terms, corresponds to truncating the distribution. Let *p_k_* be this threshold, so that the haplotypes removed are those at frequencies *p_k_*_+1_, *p_k_*_+2_, …, *p_n_*. The resulting truncated quasispecies will show haplotype frequencies resulting from normalization of the remaining haplotype frequencies, (*p*_1_, *p*_2_, …, *p_k_*), as described in Equation (8),

(8) $$\Pi^{\prime }=\left(p_{1},p_{2},\ldots ,p_{k}\right)/\overset{k}{\underset{i=1}{\sum }}p_{i}$$

where *p_i_* are the frequencies calculated from read counts before the filter. In this manner, the original frequencies are simply re-scaled. The truncation represents a loss of information, in the sense that below the error level, whatever it may be, there are authentic haplotypes that would equally be rejected.

### 4.6. Sample Size Dependence

Diversity indices are dependent to a varying extent on the sample size [4], and this dependence has to be taken into account when comparing values from different samples. In the present study, we used bootstrap to correct differences due to sample size. From the set of samples to be compared, the minimum coverage was taken as the sample size reference. Each sample then underwent 1000 bootstrap cycles (i.e., sampling with repositioning), taking the frequencies of all haplotypes in the sample as the probabilities, and the minimum coverage in the set of samples to be compared as the sample size. In each cycle, each diversity index was calculated from the resulting bootstrap sampling. At the end of the 1000 cycles, averages and standard deviations were computed for each diversity index in the study. In this way all diversity index values were referenced to the same sample size (coverage).

In previous research, we suggested a faster alternative to this process, calling it down-sampling with fringe trimming [3,4]. Although it provided good correction of sample size bias, it resulted in a loss of information that can be critical in some situations.

### 4.7. Distance between Quasispecies, Quasispecies Dendrograms, and Multidimensional Scale Plots (MDS)

A quasispecies can be seen as a genetic population, and the methods used to study diversification in a genetic population can also be used to determine the distance or dissimilarity between different quasispecies. There are several useful methods to quantify distance. When examining the Hamming distance, or any genetic distance, between pairs of haplotypes in two populations (quasispecies), the nucleotide divergence (Da) formula by Matoshi Nei [51] measures the net genetic distance between them, correcting the full genetic distance by subtracting their mean intra-population genetic diversity. This same method can be applied to calculate the inter-quasispecies phenotype distance, in this case substituting the matrices of genetic distances between pairs of haplotypes for the matrices of distances between amino acid haplotypes. The distance between proteins can be computed by the method of Grishin [52] to obtain dissimilarities between proteins from substitution matrices, such as PAM or BLOSUM. Alternatively they can be computed directly from matrices of distances between pairs of amino acids, using methods such as that of Fitch [53], or Grantham [54]. When the quasispecies are closely related, as in the HEV follow-up study here, an alternative type of distance or dissimilarity of interest can be obtained directly from the haplotype frequencies of the two quasispecies, regardless of the genetic distance between them, by applying the method of Yue—Clayton [55]. This method can also be applied to the quasispecies fitness fractions introduced above. 

The distances or dissimilarities obtained can be used to plot quasispecies dendrograms or trees, or multidimensional scaling maps, to help visualize how the quasispecies in a study are related.

### 4.8. Software and Statistics

All computations were done in R (v4.0.3) [56] with in-house scripts, using the Biostrings [57], ShortRead [58], and QSutils [59] packages from Bioconductor [60], as well as ape [61], tidyverse [62], and ggplot2 [63].

## Figures and Tables

**Table 1:** Median (Interquartile range) values of each fraction by treatment condition over the three amplicons.

	Master	Emerging	RHL_1_0.1	RHL_0.1
Control	0.436 (0.0030)	0.283 (0.0566)	0.085 (0.0232)	0.197 (0.0304)
FPV	0.184 (0.0541)	0.298 (0.111)	0.186 (0.0453)	0.290 (0.0519)
RBV	0.211 (0.0235)	0.331 (0.0586)	0.160 (0.0404)	0.293 (0.0366)
SOF	0.680 (0.0403)	0.081 (0.0577)	0.064 (0.0142)	0.158 (0.0348)

**Table 2:** Follow-up data, with dates and intervals, viral loads expressed as logarithms, and clinical observations. EOT, end of treatment.

Date (Y-M-D)	Interval (Days)	Days Since Diagnosis	Sample ID	LogVL	Observations
2018-05-18	0	0		5.91	Diagnosis
2018-05-23	5	5	S01	5.87	
2018-07-31	69	74	S03	4.60	Ribavirin 600 mg
2018-08-28	28	102	S04	4.60	
2018-10-23	56	158		1.54	EOT
2018-11-20	28	186	S06	3.04	Relapse
2019-01-18	59	245	S08	5.18	
2019-10-02	257	502	S10	6.43	Ribavirin 800 mg
2019-10-30	28	530	S12	4.62	
2019-11-27	28	558	S14	3.18	
2019-12-18	21	579	S16	2.04	
2020-03-18	91	670	S17	3.04	Ribavirin 1000 mg
2020-04-20	33	703	S18	3.40	
2020-05-20	30	733	S20	3.68	EOT
2020-06-17	28	761		4.45	Relapse
2021-06-02	350	1111	S24	6.28	

## Data Availability

The genomic nucleotide sequences included in this study have been submitted in the GENBank repository database as BioProject ID PRJNA876218.

## Supplementary Materials

The following supporting information can be downloaded at: https://www.mdpi.com/article/10.3390/ijms232314654/s1.

## References

[1] Domingo E., Escarmis C., Lazaro E., Manrubia S.C. (2005). Quasispecies dynamics and RNA virus extinction. Virus Res..

[2] Domingo E. (2016). Virus as Populations. Composition, Complexity, Dynamics and Biological Implications. Virus as Populations.

[3] Gregori J., Salicru M., Domingo E., Sanchez A., Esteban J.I., Rodriguez-Frias F., Quer J. (2014). Inference with viral quasispecies diversity indices: Clonal and NGS approaches. Bioinformatics.

[4] Gregori J., Perales C., Rodriguez-Frias F., Esteban J.I., Quer J., Domingo E. (2016). Viral quasispecies complexity measures. Virology.

[5] Vellas C., Del Bello A., Debard A., Steinmeyer Z., Tribaudeau L., Ranger N., Jeanne N., Martin-Blondel G., Delobel P., Kamar N. (2022). Influence of treatment with neutralizing monoclonal antibodies on the SARS-CoV-2 nasopharyngeal load and quasispecies. Clin. Microbiol. Infect. Off. Publ. Eur. Soc. Clin. Microbiol. Infect. Dis..

[6] Perales C., Martín V., Ruiz-Jarabo C.M., Domingo E. (2005). Monitoring sequence space as a test for the target of selection in viruses. J. Mol. Biol..

[7] Gregori J., Soria M.E., Gallego I., Guerrero-Murillo M., Esteban J.I., Quer J., Perales C., Domingo E. (2018). Rare haplotype load as marker for lethal mutagenesis. PLoS ONE.

[8] Perales C., Gallego I., de Ávila A.I., Soria M.E., Gregori J., Quer J., Domingo E. (2019). The increasing impact of lethal mutagenesis of viruses. Future Med. Chem..

[9] Nakano K., Shiroma A., Shimoji M., Tamotsu H., Ashimine N., Ohki S., Shinzato M., Minami M., Nakanishi T., Teruya K. (2017). Advantages of genome sequencing by long-read sequencer using SMRT technology in medical area. Hum. Cell.

[10] Bull R.A., Eltahla A.A., Rodrigo C., Koekkoek S.M., Walker M., Pirozyan M.R., Betz-Stablein B., Toepfer A., Laird M., Oh S. (2016). A method for near full-length amplification and sequencing for six hepatitis C virus genotypes. BMC Genom..

[11] Dilernia D.A., Chien J.T., Monaco D.C., Brown M.P.S., Ende Z., Deymier M.J., Yue L., Paxinos E.E., Allen S., Tirado-Ramos A. (2015). Multiplexed highly-accurate DNA sequencing of closely-related HIV-1 variants using continuous long reads from single molecule, real-time sequencing. Nucleic Acids Res..

[12] Stoler N., Nekrutenko A. (2021). Sequencing error profiles of Illumina sequencing instruments. NAR Genom. Bioinforma..

[13] Amarasinghe S.L., Su S., Dong X., Zappia L., Ritchie M.E., Gouil Q. (2020). Opportunities and challenges in long-read sequencing data analysis. Genome Biol..

[14] Wenger A.M., Peluso P., Rowell W.J., Chang P.C., Hall R.J., Concepcion G.T., Ebler J., Fungtammasan A., Kolesnikov A., Olson N.D. (2019). Accurate circular consensus long-read sequencing improves variant detection and assembly of a human genome. Nat. Biotechnol..

[15] Hill M.O. (1973). Diversity and Evenness: A Unifying Notation and Its Consequences. Ecology.

[16] Carcereny A., Martínez-Velázquez A., Bosch A., Allende A., Truchado P., Cascales J., Romalde J.L., Lois M., Polo D., Sánchez G. (2021). Monitoring Emergence of the SARS-CoV-2 B.1.1.7 Variant through the Spanish National SARS-CoV-2 Wastewater Surveillance System (VATar COVID-19). Environ. Sci. Technol..

[17] Twist Synthetic SARS-CoV-2 RNA Control 2 MN908947.3. https://www.twistbioscience.com/es/resources/product-sheet/twist-synthetic-sars-cov-2-rna-controls.

[18] Cubero M., Gregori J., Esteban J.I., Garcia-Cehic D., Bes M., Perales C., Domingo E., Rodriguez-Frias F., Sauleda S., Casillas R. (2014). Identification of host and viral factors involved in a dissimilar resolution of a hepatitis C virus infection. Liver Int..

[19] Baccam P., Thompson R.J., Fedrigo O., Carpenter S., Cornette J.L. (2001). PAQ: Partition Analysis of Quasispecies. Bioinformatics.

[20] Töpfer A., Marschall T., Bull R.A., Luciani F., Schönhuth A., Beerenwinkel N. (2014). Viral quasispecies assembly via maximal clique enumeration. PLoS Comput. Biol..

[21] Skums P., Zelikovsky A., Singh R., Gussler W., Dimitrova Z., Knyazev S., Mandric I., Ramachandran S., Campo D., Jha D. (2018). QUENTIN: Reconstruction of disease transmissions from viral quasispecies genomic data. Bioinformatics.

[22] Ahn S., Ke Z., Vikalo H. (2018). Viral quasispecies reconstruction via tensor factorization with successive read removal. Bioinformatics.

[23] Henningsson R., Moratorio G., Bordería A.V., Vignuzzi M., Fontes M. (2019). DISSEQT-DIStribution-based modeling of SEQuence space Time dynamics. Virus Evol..

[24] Beerenwinkel N., Zagordi O. (2011). Ultra-deep sequencing for the analysis of viral populations. Curr.Opin.Virol..

[25] Lorenzo-Redondo R., Delgado S., Morán F., Lopez-Galindez C. (2014). Realistic three dimensional fitness landscapes generated by self organizing maps for the analysis of experimental HIV-1 evolution. PLoS ONE.

[26] Delgado S., Perales C., García-Crespo C., Soria M.E., Gallego I., de Ávila A.I., Martínez-González B., Vázquez-Sirvent L., López-Galíndez C., Morán F. (2021). A Two-Level, Intramutant Spectrum Haplotype Profile of Hepatitis C Virus Revealed by Self-Organized Maps. Microbiol. Spectr..

[27] Gregori J., Cortese M.F., Piñana M., Campos C., Garcia-Cehic D., Andrés C., Abril J.F., Codina M.G., Rando A., Esperalba J. (2021). Host-dependent editing of SARS-CoV-2 in COVID-19 patients. Emerg. Microbes Infect..

[28] Martínez-González B., Soria M.E., Vázquez-Sirvent L., Ferrer-Orta C., Lobo-Vega R., Mínguez P., de la Fuente L., Llorens C., Soriano B., Ramos-Ruíz R. (2022). SARS-CoV-2 Mutant Spectra at Different Depth Levels Reveal an Overwhelming Abundance of Low Frequency Mutations. Pathogens.

[29] De Avila A.I., Gallego I., Soria M.E., Gregori J., Quer J., Ignacio Esteban J., Rice C.M., Domingo E., Perales C. (2016). Lethal mutagenesis of hepatitis C virus induced by favipiravir. PLoS ONE.

[30] Perales C., Agudo R., Tejero H., Manrubia S.C., Domingo E. (2009). Potential benefits of sequential inhibitor-mutagen treatments of RNA virus infections. PLoS Pathog..

[31] Githaka J.M. (2022). Molnupiravir Does Not Induce Mutagenesis in Host Lung Cells during SARS-CoV-2 Treatment. Bioinform. Biol. Insights.

[32] Gordon C.J., Tchesnokov E.P., Schinazi R.F., Götte M. (2021). Molnupiravir promotes SARS-CoV-2 mutagenesis via the RNA template. J. Biol. Chem..

[33] Cameron C.E., Castro C. (2001). The mechanism of action of ribavirin: Lethal mutagenesis of RNA virus genomes mediated by the viral RNA-dependent RNA polymerase. Curr. Opin. Infect. Dis..

[34] Dietz J., Schelhorn S.-E., Fitting D., Mihm U., Susser S., Welker M.-W., Füller C., Däumer M., Teuber G., Wedemeyer H. (2013). Deep sequencing reveals mutagenic effects of ribavirin during monotherapy of hepatitis C virus genotype 1-infected patients. J. Virol..

[35] Cuevas J.M., González-Candelas F., Moya A., Sanjuán R. (2009). Effect of ribavirin on the mutation rate and spectrum of hepatitis C virus in vivo. J. Virol..

[36] Todt D., Meister T.L., Steinmann E. (2018). Hepatitis E virus treatment and ribavirin therapy: Viral mechanisms of nonresponse. Curr. Opin. Virol..

[37] Todt D., Gisa A., Radonic A., Nitsche A., Behrendt P., Suneetha P.V., Pischke S., Bremer B., Brown R.J.P., Manns M.P. (2016). In vivo evidence for ribavirin-induced mutagenesis of the hepatitis E virus genome. Gut.

[38] Debing Y., Gisa A., Dallmeier K., Pischke S., Bremer B., Manns M., Wedemeyer H., Suneetha P.V., Neyts J. (2014). A mutation in the hepatitis E virus RNA polymerase promotes its replication and associates with ribavirin treatment failure in organ transplant recipients. Gastroenterology.

[39] Lhomme S., Kamar N., Nicot F., Ducos J., Bismuth M., Garrigue V., Petitjean-Lecherbonnier J., Ollivier I., Alessandri-Gradt E., Goria O. (2015). Mutation in the Hepatitis E Virus Polymerase and Outcome of Ribavirin Therapy. Antimicrob. Agents Chemother..

[40] Debing Y., Ramière C., Dallmeier K., Piorkowski G., Trabaud M.-A., Lebossé F., Scholtès C., Roche M., Legras-Lachuer C., de Lamballerie X. (2016). Hepatitis E virus mutations associated with ribavirin treatment failure result in altered viral fitness and ribavirin sensitivity. J. Hepatol..

[41] ARTIC Network. https://artic.network/ncov-2019.

[42] Andrés C., Garcia-Cehic D., Gregori J., Piñana M., Rodriguez-Frias F., Guerrero-Murillo M., Esperalba J., Rando A., Goterris L., Codina M.G. (2020). Naturally occurring SARS-CoV-2 gene deletions close to the spike S1/S2 cleavage site in the viral quasispecies of COVID19 patients. Emerg. Microbes Infect..

[43] Gallego I., Gregori J., Soria M.E., Garcia-Crespo C., Garcia-Alvarez M., Gomez-Gonzalez A., Valiergue R., Gomez J., Esteban J.I., Quer J. (2018). Resistance of high fitness hepatitis C virus to lethal mutagenesis. Virology.

[44] Gallego I., Sheldon J., Moreno E., Gregori J., Quer J., Esteban J.I., Rice C.M., Domingo E., Perales C. (2016). Barrier-independent, fitness-associated differences in sofosbuvir efficacy against hepatitis c virus. Antimicrob. Agents Chemother..

[45] Marukian S., Jones C.T., Andrus L., Evans M.J., Ritola K.D., Charles E.D., Rice C.M., Dustin L.B. (2008). Cell culture-produced hepatitis C virus does not infect peripheral blood mononuclear cells. Hepatology.

[46] Perales C., Beach N.M., Gallego I., Soria M.E., Quer J., Esteban J.I., Rice C., Domingo E., Sheldon J. (2013). Response of hepatitis C virus to long-term passage in the presence of alpha interferon: Multiple mutations and a common phenotype. J. Virol..

[47] Moreno E., Gallego I., Gregori J., Lucía-Sanz A., Soria M.E., Castro V., Beach N.M., Manrubia S., Quer J., Esteban J.I. (2017). Internal Disequilibria and Phenotypic Diversification during Replication of Hepatitis C Virus in a Noncoevolving Cellular Environment. J. Virol..

[48] Magoc T., Salzberg S.L. (2011). FLASH: Fast length adjustment of short reads to improve genome assemblies. Bioinformatics.

[49] Chao A., Gotelli N.J., Hsieh T.C., Sander E.L., Ma K.H., Colwell R.K., Ellison A.M. (2014). Rarefaction and extrapolation with Hill numbers: A framework for sampling and estimation in species diversity studies. Ecol. Monogr..

[50] Gregori J., Esteban J.I., Cubero M., Garcia-Cehic D., Perales C., Casillas R., Alvarez-Tejado M., Rodriguez-Frias F., Guardia J., Domingo E. (2013). Ultra-deep pyrosequencing (UDPS) data treatment to study amplicon HCV minor variants. PLoS ONE.

[51] Nei M. (1987). Molecular Evolutionary Genetics.

[52] Grishin V.N., Grishin N. (2002). V Euclidian space and grouping of biological objects. Bioinformatics.

[53] Fitch W.M. (1966). An improved method of testing for evolutionary homology. J. Mol. Biol..

[54] Grantham R. (1974). Amino acid difference formula to help explain protein evolution. Science.

[55] Yue J.C., Clayton M.K. (2005). A Similarity Measure Based on Species Proportions. Commun. Stat.-Theory Methods.

[56] R Core Team (2019). R: A Language and Environment for Statistical Computing.

[57] Pages H., Aboyoun P., Gentleman R., DebRoy S. Biostrings: String Objects Representing Biological Sequences, and Matching Algorithms, R package 2.38.4; 2012. https://bioc.ism.ac.jp/packages/3.2/bioc/html/Biostrings.html.

[58] Morgan M., Anders S., Lawrence M., Aboyoun P., Pages H., Gentleman R. (2009). ShortRead: A bioconductor package for input, quality assessment and exploration of high-throughput sequence data. Bioinformatics.

[59] Guerrero-Murillo M., Gregori J. QSutils: Quasispecies Diversity. R Package Version 1.0.0. https://bioconductor.org/packages/release/bioc/html/QSutils.html_2018.

[60] Gentleman R.C., Carey V.J., Bates D.M., Bolstad B., Dettling M., Dudoit S., Ellis B., Gautier L., Ge Y., Gentry J. (2004). Bioconductor: Open software development for computational biology and bioinformatics. Genome Biol..

[61] Paradis E., Schliep K. (2019). ape 5.0: An environment for modern phylogenetics and evolutionary analyses in R. Bioinformatics.

[62] Wickham H. (2019). Welcome to Master the Tidyverse. J. Open Source Softw..

[63] Valero-Mora P.M. (2010). ggplot2: Elegant Graphics for Data Analysis. J. Stat. Soft. Book Rev..

