# Supplementary material for: Quasispecies Fitness Partition to Characterize the Molecular Status of a Viral Population. Negative Effect of Early Ribavirin Discontinuation in a Chronically Infected HEV Patient
Source: Int J Mol Sci. 2022 Nov 24;23(23):14654. doi: 10.3390/ijms232314654 (PMC9739305; doi:10.3390/ijms232314654)
Supplement: Supplementary file 1 [file ijms-23-14654-s001.zip › ijms-1982579-supplementary.pdf]

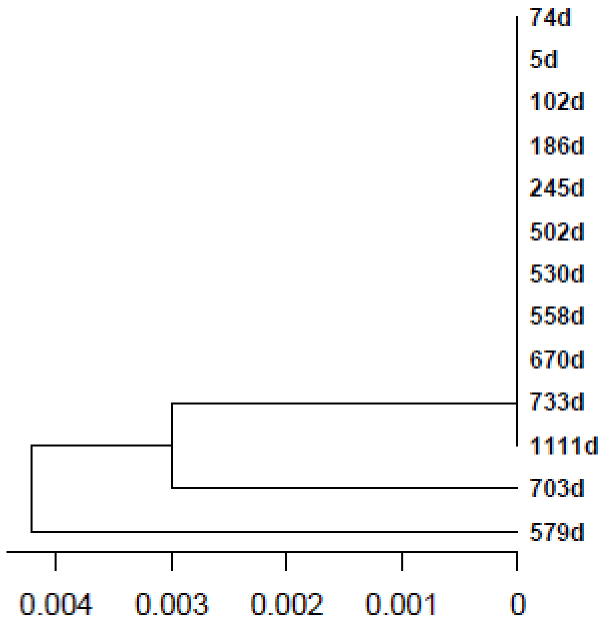

(a)

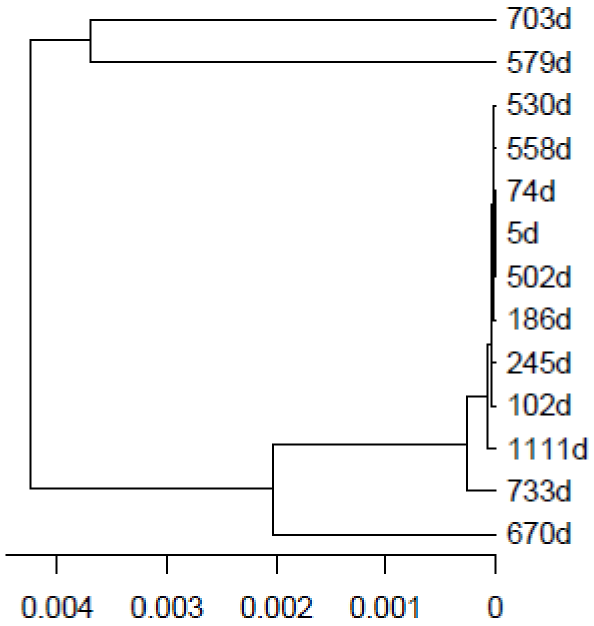

(b)

Figure S1: UPGMA tree of the master phenotypes based on Grantham amino acid distances (a), and quasispecies tree based on the Da population distances (b), taking the top 20 phenotypes in each sample.

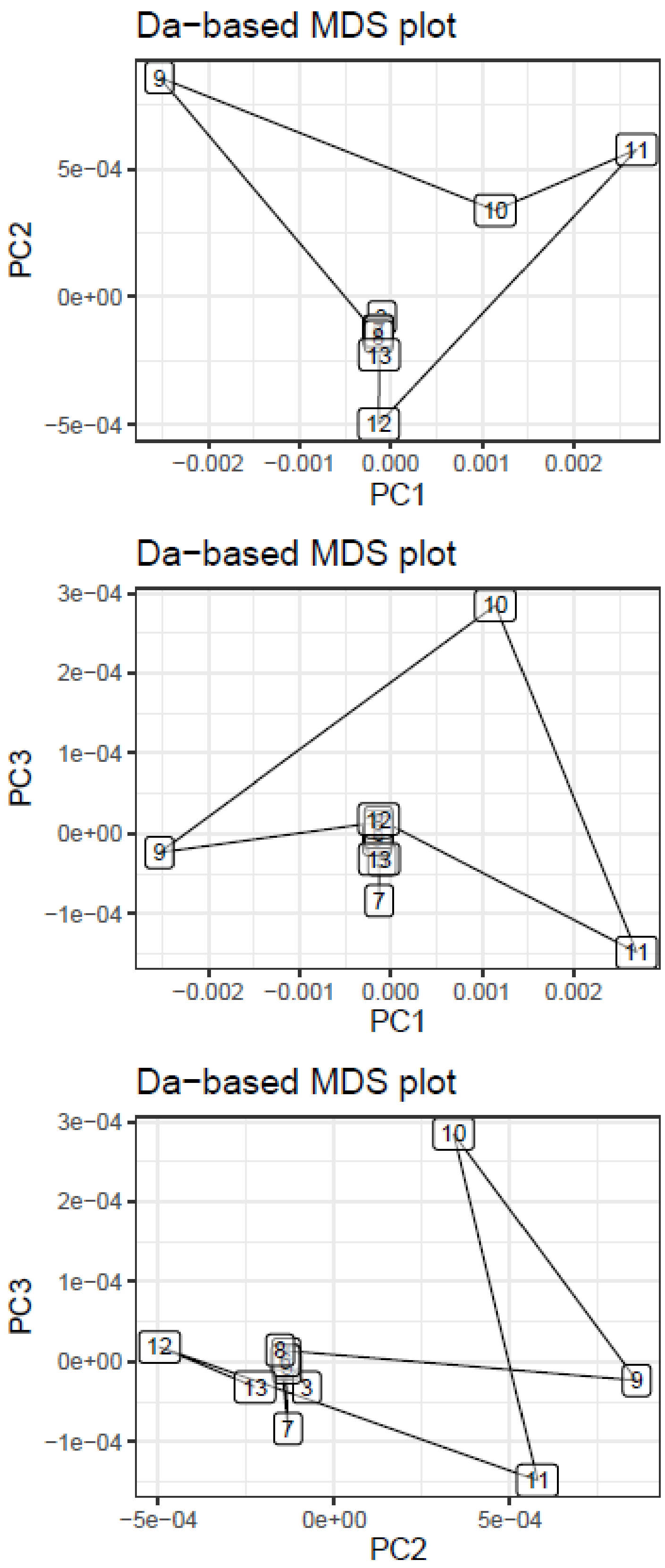

Figure S2: Multidimensional scaling plot of phenotype distances between quasispecies, taking the top 20 phenotypes in each sample.

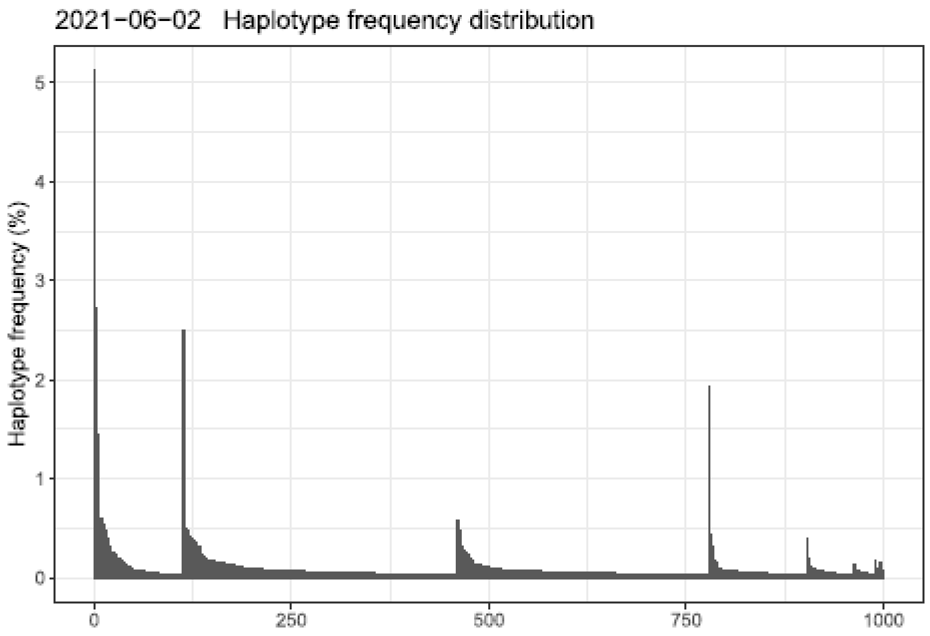

Figure S3: Montserrat plot with the distribution of the 1000 most abundant haplotypes in the last sample (1111 days since diagnosis, 2 June 2020).

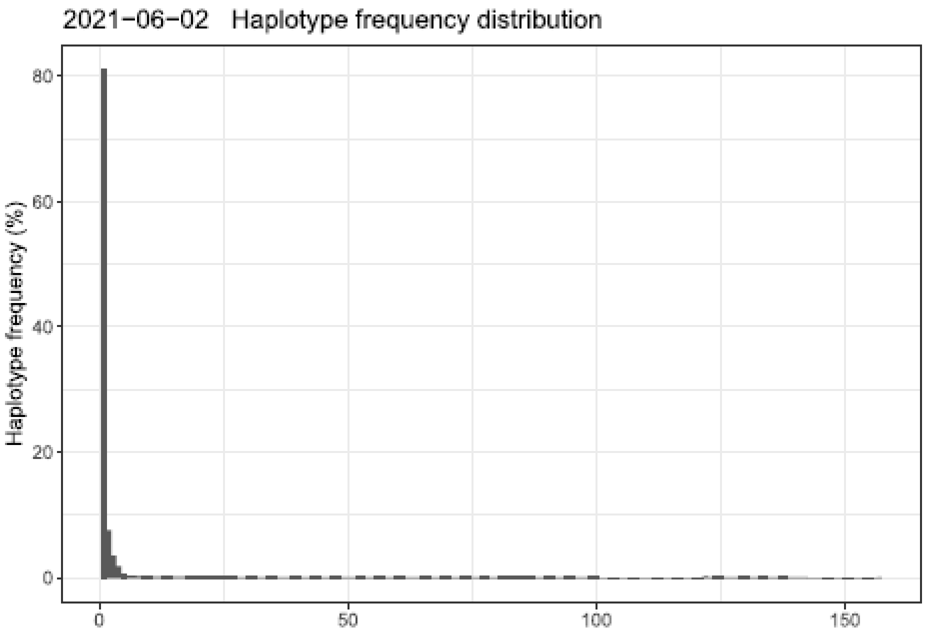

Figure S4: Montserrat plot with the distribution of all phenotypes in the last sample (1111 days since diagnosis, 2 June 2020).

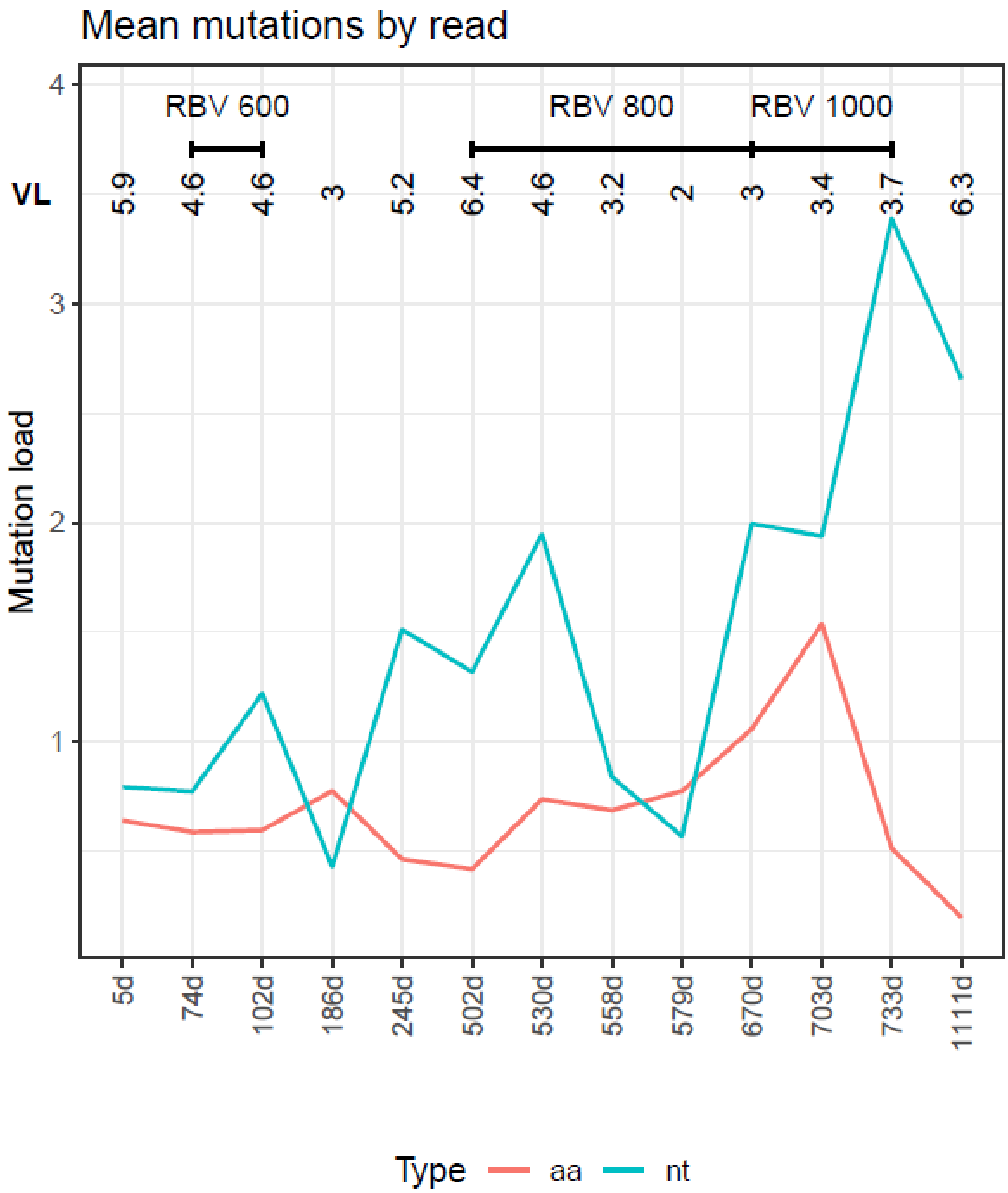

Figure S5: Mean number of substitutions per read with respect to the master haplotype, at the nucleotide level (turquoise), and mean number of mutations per read with respect to the master phenotype at the amino acid level (orange).
